# Тяжелые костные осложнения первичного гиперпаратиреоза у молодого пациента с верифицированной мутацией в гене <i>MEN1</i>

**DOI:** 10.14341/probl12864

**Published:** 2022-02-18

**Authors:** А. К. Еремкина, Д. В. Сазонова, Е. Е. Бибик, А. З. Шейхова, А. В. Хайриева, Ю. В. Буклемишев, Н. Г. Мокрышева

**Affiliations:** Национальный медицинский исследовательский центр эндокринологии; Национальный медицинский исследовательский центр эндокринологии; Национальный медицинский исследовательский центр эндокринологии; Национальный медицинский исследовательский центр эндокринологии; Национальный медицинский исследовательский центр эндокринологии; Национальный медицинский исследовательский центр травматологии и ортопедии им. Н.Н. Приорова; Национальный медицинский исследовательский центр эндокринологии

**Keywords:** синдром множественных эндокринных неоплазий типа 1, первичный гиперпаратиреоз, снижение минеральной плотности костей, менин, фиброзно-кистозный остеит, патологические переломы костей

## Abstract

Синдром множественных эндокринных неоплазий 1 типа (МЭН1) – это редкое наследственное заболевание, проявляющееся различными комбинациями из более чем 20 эндокринных и неэндокринных опухолей. К сожалению, ни одна из описанных мутаций MEN1 не была связана со специфическим клиническим фенотипом даже в пределах одной семьи. Синдром МЭН1 – самая частая причина наследственного первичного гиперпаратиреоза (ПГПТ), пенетрантность заболевания которого превышает 50% к 20 годам и достигает 95% к возрасту 40 лет.  К одному из ключевых симптомов при ПГПТ, как при спорадической, так и наследственной форме заболевания, относится поражение костной ткани. На момент постановки диагноза при ПГПТ/МЭН1, минеральная плотность ткани, как правило, ниже в сравнении со спорадической формой ПГПТ. Это может быть обусловлено как избыточной секрецией паратиреоидного гормона в период набора пика костной массы, сопутствующими компонентами синдрома, большим объемом оперативного лечения, так и непосредственным влиянием мутации в гене менина на костное ремоделирование. В представленном клиническом наблюдении мы описываем молодого пациента с редкой мутацией гена MEN1 и тяжелым течением кистозно-фиброзного остеита вследствие ПГПТ. Диагноз ПГПТ был установлен спустя 5 лет от первых костных осложнений и повторных ортопедических вмешательств. После успешного хирургического лечения ПГПТ наблюдался синдром «голодных костей», а через 6 месяцев, на фоне терапии препаратами витамина D и карбоната кальция, отмечается положительная динамика в виде прироста минеральной плотности костной ткани в осевых отделах скелета.

## Abstract (en)

Multiple endocrine neoplasia type 1 syndrome (MEN1) is a rare inherited disorder that can include combinations of more than 20 endocrine and non-endocrine tumors. Unfortunately, none of the described MEN1 mutations has been associated with a peculiar clinical phenotype, even within members of the same family, thus a genotype-to-phenotype correlation does not exist. MEN1 syndrome is the most common cause of hereditary primary hyperparathyroidism (PHPT), the disease penetrance of which exceeds 50% by the age of 20 and reaches 95% by the age of 40. At the same time, PHPT with hyperplasia or adenomas of the parathyroid glands (PTG) is the most distinctive manifestation of the MEN1 syndrome. One of the main symptoms of PHPT, both in sporadic and hereditary forms of the disease, is bone damage. At the time of diagnosis in PHPT/MEN1, the bone mineral density is generally lower in comparison with the sporadic form of PHPT. This may be due to excessive secretion of parathyroid hormone during the period of peak bone mass, concomitant components of the syndrome, extended surgical treatment, and the direct effect of a mutation in the menin gene on bone remodeling. This clinical case describes a young patient with severe bone complications of PHPT and uncertain rare MEN1 mutation. PHPT was diagnosed five years later from the first onset of bone complications and repeated orthopedic operations. There was the «hungry bones» syndrome after successful surgery of PHPT, which was managed with vitamin D and calcium carbonate supplementation and there is a positive dynamic in increased bone mineral density in the main skeleton after 6 months.

## АКТУАЛЬНОСТЬ

Синдром множественных эндокринных неоплазий 1 типа (МЭН1) (OMIM ID #131100) — аутосомно-доминантное заболевание, в классическом варианте характеризуется сочетанным развитием опухолей околощитовидной железы (90%), островкового аппарата поджелудочной железы (30−70%) и аденогипофиза (30−40%). Реже диагностируются опухоли надпочечников, нейроэндокринные новообразования (НЭН) тимуса, легких и желудочно-кишечного тракта, ангиофибромы, коллагеномы, липомы и другие образования. Большинство новообразований в рамках синдрома являются доброкачественными, клиническая картина заболевания, как правило, обусловлена гормональной гиперсекрецией или проявлением «масс-эффекта», однако сохраняется высокий риск злокачественной прогрессии этих опухолей [1]. Ожидаемая продолжительность жизни пациентов с синдромом МЭН1 ниже по сравнению с общей популяцией и составляет около 55 лет. Наиболее частой причиной смерти становятся дуодено-панкреатические НЭН [2].

В большинстве исследований сообщается о предполагаемой распространенности заболевания около 1–3 случаев на 100 000 человек [3]. Причина развития данного синдрома — мутация в гене MEN1, продуктом экспрессии которого является 610-аминокислотный белок менин, регулирующий различные функции клеточного и геномного гомеостаза. Менин модулирует активность ингибиторов клеточного цикла, на ядерном уровне инактивирует факторы транскрипции и участвует в процессах репарации ДНК. Данный белок экспрессируется во всех тканях и преимущественно располагается в ядре. Супрессивный эффект менина достигается за счет его взаимодействия с различными доменами — гистонмодифицирующими ферментами (MLL1, EZH2 и HDAC), факторами транскрипции (JunD, NF-κB, PPARγ, VDR) и другими белковыми комплексами (АР-1, Smad). На сегодняшний день описано более 1500 независимых соматических и герминативных мутаций в кодирующих участках MEN1 [4]. Большинство из них являются инактивирующими, т.е. ассоциированы с синтезом неполноценного белка, не способного реализовать основной онкосупрессорный эффект менина. Недостаточность функционирующего менина при потере обеих аллелей MEN1 приводит к опухолевому росту.

Первичный гиперпаратиреоз (ПГПТ), как правило, становится первым проявлением синдрома (до 75%), при этом распространенность МЭН1 среди пациентов с ПГПТ варьирует от 1 до 18%.

Пенетрантность заболевания превышает 50% к 20 годам и достигает 95% к возрасту 40 лет [4]. К одному из ключевых симптомов при ПГПТ, как при спорадической, так и наследственной форме заболевания, относится поражение костной ткани, проявляющееся снижением минеральной плотности костной ткани (МПК) вплоть до остеопороза с низкоэнергетическими переломами и фиброзно-кистозным остеитом [5]. Тем не менее в течении развития костных осложнений у пациентов с ПГПТ, ассоциированных с мутациями в гене MEN1 и, как следствие, синдромом МЭН1, наблюдается ряд отличий по сравнению со спорадической формой заболевания. Так, например, на момент постановки диагноза при ПГПТ/МЭН1 у большинства пациентов МПК ниже [6][7]. Также важно отметить, что при остеопорозе, являющемся следствием спорадического ПГПТ, набор костной массы после удаления аденомы околощитовидной железы происходит относительно быстрыми темпами; в связи с этим в первые несколько лет после вмешательства пациентам даже не назначается терапия остеопороза [8]. Темпы же набора костной массы после паратиреоидэктомии (ПТЭ) у пациентов с ПГПТ/МЭН1 могут быть существенно ниже [9].

Причины этих различий на сегодняшний день однозначно не установлены. С одной стороны, избыточная патологическая секреция паратиреоидного гормона (ПТГ) при манифестации МЭН1 нередко возникает в период набора пика костной массы, повышенная резорбция костной ткани приводит к ранней потере кортикального и трабекулярного вещества кости, что клинически проявляется более ранними костными осложнениями, в сравнении со спорадической формой ПГПТ. Также к дополнительному снижению МПК при ПГПТ в рамках МЭН1 могут приводить сопутствующие компоненты синдрома, например, гормонально-активные НЭН желудочно-кишечного тракта (ЖКТ) [10]. Низкие темпы набора костной массы после хирургического лечения могут быть следствием большего объема операции и высокой частоты послеоперационного гипопаратиреоза [9]. Тем не менее, согласно современным представлениям, не исключается влияние непосредственно мутации в гене менина на костное ремоделирование. Патогенетические механизмы взаимосвязи инактивации менина и разобщения костного ремоделирования активно изучаются. В разделе «Обсуждение» изложены ключевые гипотезы, объясняющие причины тяжелого остеопороза при МЭН1.

В данной статье представлено клиническое наблюдение за пациентом с редкой мутацией в гене MEN1 и тяжелым течением фиброзно-кистозного остеита вследствие ПГПТ.

## ОПИСАНИЕ СЛУЧАЯ

Впервые в возрасте 18 лет (2015 г.) во время прыжков через скакалку у пациента И. произошел низкоэнергетический перелом костей правой голени в нижней трети. Спустя полгода, при падении с велосипеда, возник перелом костей правой голени в верхней трети, консолидация перелома произошла при гипсовой фиксации. Через 3 года диагностирован патологический перелом метаэпифиза левой плечевой кости на фоне костной кисты (подтвержден рентгенологически), проведен остеосинтез штифтом с блокированием в сочетании с аутопластикой дефекта.

В связи с возникновением болевого синдрома в 2018 г. при рентгенографии выявлен патологический очаг в левой ключице: дистальный конец ключицы деформирован, «вздут», структура костной ткани неоднородна за счет участка просветления с четкими контурами 13×42 мм, кортикальный слой истончен. Данное образование было расценено как киста левой ключицы. В связи с прогрессированием болевого синдрома и ограничением объема движений в левом плечевом суставе в 2019 г. выполнена краевая резекция дистального конца левой ключицы с аутопластикой дефекта из крыла подвздошной кости.

В июле 2020 г. после низкоэнергетической травмы (падения) при рентгенологическом исследовании правого коленного сустава выявлены множественные очаги деструкции костей, образующих коленный сустав. Данные изменения расценены как множественные кисты (рис. 1, а, б). По данным магнитно-резонансной томографии (МРТ) также подтверждены признаки множественных кист в дистальных метаэпифизах бедренных и проксимальных отделах большеберцовых костей с обеих сторон, справа — с разрушением кортикального слоя кости (рис. 2, а, б).

Для уточнения генеза костных нарушений и исключения метастатического поражения скелета по месту жительства проведена сцинтиграфия с Пирфотех 99mTc всего тела, по результатам которой наблюдались генерализованные остеобластические и остеолитические процессы в костной ткани (рис. 3). Пациент был осмотрен онкологом, проведена биопсия кистозных изменений правой большеберцовой и бедренной костей, по результатам которой получены фрагменты фиброзной ткани с включениями небольших очагов реактивного костеобразования, эпителиальная выстилка не определялась. Гистологически верифицированы костные кисты. Лабораторное исследование состояния фосфорно-кальциевого обмена в этот период времени не проводилось.

В рамках заочной телемедицинской консультации в ФГБУ «НМИЦ травматологии и ортопедии им.  Н.Н. Приорова» Минздрава России пациенту было рекомендовано дообследование, включающее биохимические и гормональные показатели минерального обмена, МРТ в специальном режиме диффузионно-взвешенной визуализации с подавлением фонового сигнала с целью исключения распространенного онкологического процесса, рентгеновская денситометрия основных отделов скелета, консультация генетика для исключения генетически обусловленных поражений скелета. Рекомендовано соблюдение ортопедического режима: ходьба при помощи костылей, внешняя фиксация в гипсе/ортезе.

При лабораторном исследовании зафиксирована гиперкальциемия (кальций общий — 2,7 ммоль/л, кальций ионизированный — 1,65 ммоль/л), гиперкальциурия (кальций суточной мочи — 14,2 ммоль/сут), повышение уровня ПТГ до 45 пмоль/л (референсные значения (РИ) 1,45–10) на фоне тяжелого дефицита витамина D (25(OH)D — 9 нг/мл), что соответствовало диагнозу ПГПТ. Кроме того, отмечено превышение референсных границ маркеров костной резорбции: щелочная фосфатаза 346 Ед/л (РИ до 270), С-концевой телопептид коллагена 1 типа — 1,490 нг/мл (РИ 0,087–1,200), N-терминальный пропептид проколлагена 1 типа (Р1NP) — 148 нг/мл (РИ 15–115), остеокальцин — 68 нг/мл (24–70). По результатам остеоденситометрии поясничного отдела позвоночника максимальное снижение МПК составило -1,15 SD по Z-критерию.

При МР-исследовании выявлены кистозные изменения правой подвздошной и левой лонной костей. А также визуализировано округлое образование каудальнее левой доли щитовидной железы, паратрахеально слева, гиперинтенсивного сигнала на Т2-взвешенных изображениях (Т2-ВИ) и умеренно гипоинтенсивного на Т1-ВИ, несколько неоднородной структуры, размерами 1,5×1,9 см. В результате обследования исключено наличие наследственной остеодистрофии и других метаболических остеопатий.

В период прохождения обследования, несмотря на рекомендации, пациент не соблюдал ортопедический режим и при приседании отметил резкую боль в левом бедре, при обследовании диагностирован закрытый патологический надмыщелковый перелом левой бедренной кости. Проведена открытая репозиция перелома, металлоостеосинтез пластинами с аллопластикой дефекта (рис. 4).

После повторной телемедицинской консультации в ФГБУ «НМИЦ травматологии и ортопедии им. Н.Н. Приорова» Минздрава России заподозрен ПГПТ, рекомендованы проведение топической диагностики и направление пациента для дальнейшего лечения в специализированный эндокринологический стационар.

По данным УЗИ щитовидной и околощитовидных желез патологических изменений не выявлялось. Дополнительно проводилась однофотонная эмиссионная компьютерная томография (ОФЭКТ) с Технетрилом 99mTc (МИБИ), в тиреоидную фазу (через 60 мин) в переднем средостении, за грудиной, на расстоянии 18 мм от нижнего полюса левой доли, визуализировано образование 19,4×13,8 мм. В паратиреоидную фазу (через 2 ч) определялись околощитовидные железы (ОЩЖ) в виде очагов округлой формы, размером до 2,0 мм, с повышенным включением Технетрила Tc-99m в проекции левой доли — 4, правой доли — 2; дополнительно сохранялся очаг гиперфиксации радиофармпрепарата в переднем средостении (рис. 5). При МСКТ, под левой долей щитовидной железы подтверждено образование неправильной формы, размерами 15×11×20 мм, нативной плотностью от 5 до 40 HU, при контрастировании максимально накапливающее контрастный препарат до 150 HU.

**Figure:**
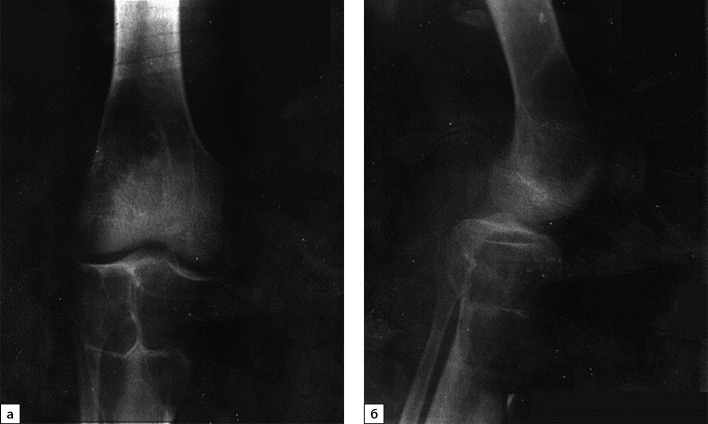
Рисунок 1. Рентгенография правого коленного сустава с литическими очагами бедренной и большеберцовой костей, 2020 г. (в прямой (а) и боковой проекциях (б)).

**Figure:**
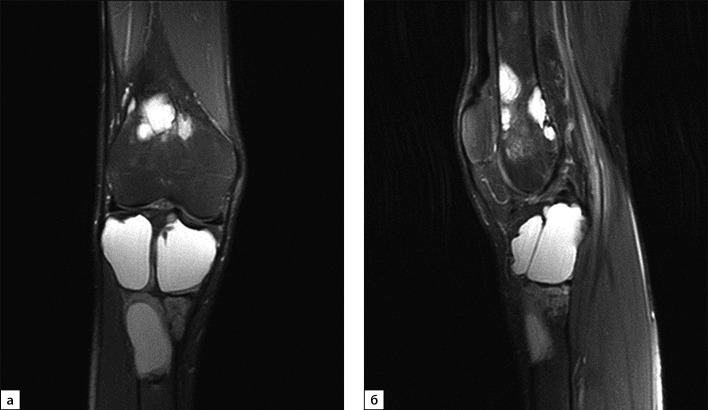
Рисунок 2. Магнитно-резонансная томография правого коленного сустава в режиме Т2, 2020 г. (в корональной (а) и сагиттальной проекциях (б)): МР-картина множественных костных кист в бедренной и большеберцовой костях. Отмечается истончение кортикального слоя кости, местами вздутие.

**Figure:**
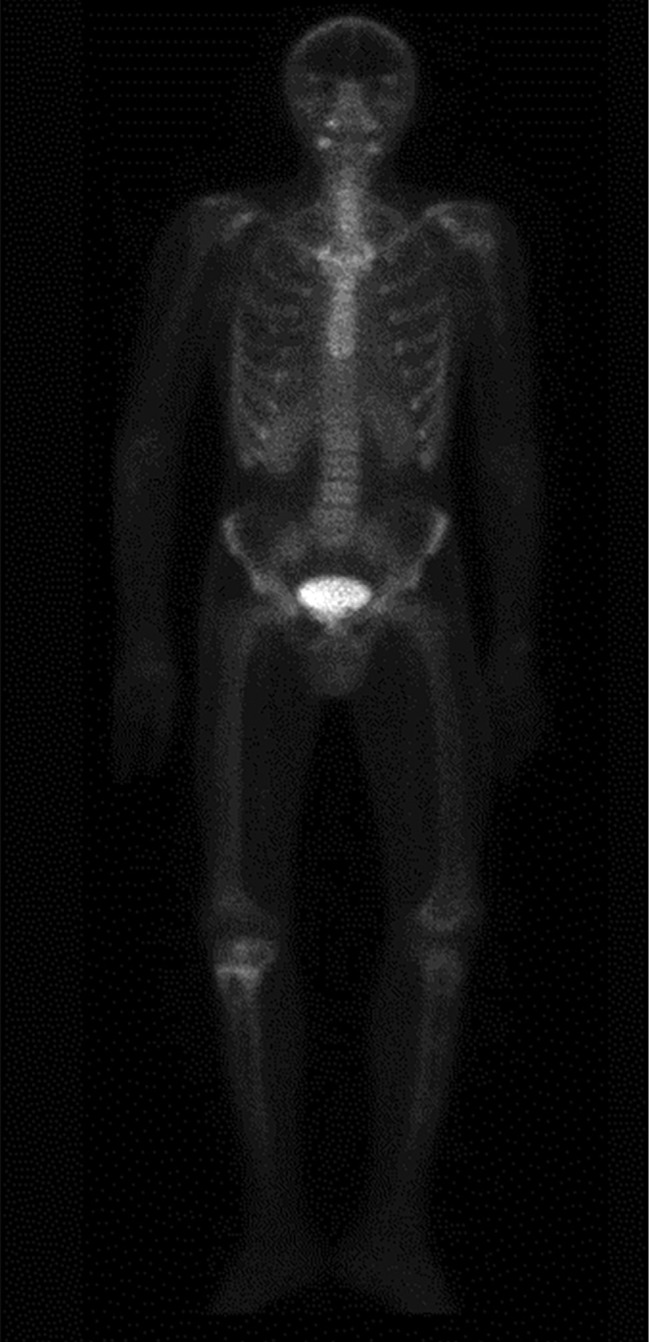
Рисунок 3. Однофотонная эмиссионная компьютерная томография с Пирфотех 99mTc всего тела: генерализованный остеобластический и остеолитический процессы в костной ткани, с наиболее выраженным проявлением в проксимальном метафизе правой большеберцовой кости.

**Figure:**
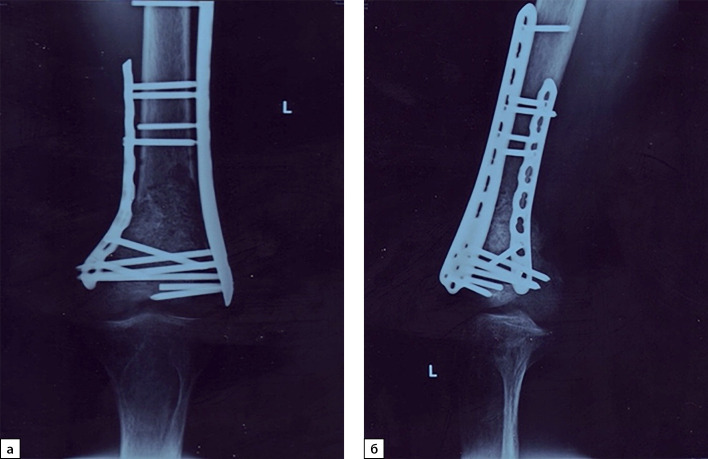
Рисунок 4. Рентгенография левого коленного сустава, 2021 г. (в прямой (а) и боковой проекциях (б)): состояние после оперативного лечения — остеосинтеза бедренной кости в нижней трети, аллопластики дефекта.

**Figure:**
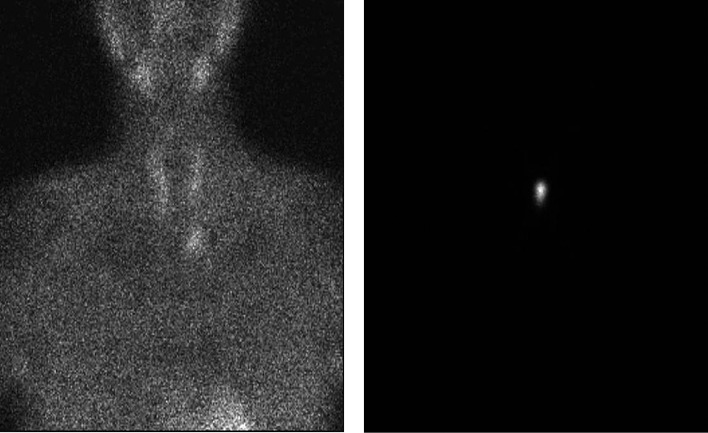
Рисунок 5. Однофотонная эмиссионная компьютерная томография с Технетрилом 99mTc (MIBI), 2021 г.: очаг гиперфиксации РФП в области левой нижней околощитовидной железы.

Для дообследования и лечения пациент И. направлен в ФГБУ «НМИЦ эндокринологии» Минздрава России.

Основными жалобами пациента при поступлении в отделение патологии околощитовидных желез были выраженные боли в коленных суставах, затрудняющие самостоятельное передвижение, спонтанные боли в левом лучезапястном суставе, в поясничном отделе позвоночника, эпизоды сильных головных болей (до 1 раза в месяц), учащенное мочеиспускание.

При осмотре отмечены дефицит массы тела (индекс массы тела 16,8 кг/м2), выраженное снижение общей мышечной массы, особенно в области бедер. Кожные покровы обычной окраски, сухие, множественные послеоперационные рубцы в местах проведения травматологических вмешательств. Выявлены грудной гиперкифоз, ограничение подвижности в правом плечевом и коленных суставах с двух сторон, при пальпации умеренная болезненность, передвижение с помощью трости. Щитовидная железа не увеличена, безболезненная, подвижная, узловые образования не пальпируются, периферические шейные лимфоузлы не увеличены.

Результаты лабораторного обследования были сопоставимы с показателями, определенными на амбулаторном этапе (табл. 1).

При УЗИ визуализировано образование левой нижней ОЩЖ размерами 2,0×1,4×1,3 см, что совпадало с данными ранее проведенных ОФЭКТ и МСКТ. С целью оценки тяжести осложнений ПГПТ выполнено УЗИ почек, выявлены признаки начального нефрокальциноза, пиелоэктазия справа, микролиты обеих почек. По результатам остеоденситометрии подтверждено снижение МПК относительно ожидаемой по возрасту в проксимальном отделе бедренной кости до -3,0 SD, в позвоночнике -1,5 SD и в лучевой кости -1,7 SD по Z-критерию.

В связи с молодым возрастом дебюта ПГПТ, для исключения наследственной формы заболевания проведено полное секвенирование гена MEN1: в 10 экзоне выявлен вариант мутации в гетерозиготном состоянии, вероятно патогенная — c.1609G> T, p.Gly537Cys (rs587780843). Данный вариант встречается в базах данных с очень низкой частотой (3 аллеля на 250 106). В литературе ранее описан не был.

Учитывая наличие генетической мутации у пациента И., его родственникам 1-й линии было рекомендовано обследование для исключения нарушений фосфорно-кальциевого обмена, патологических отклонений выявлено не было (табл. 2), семейный анамнез по эндокринологическим заболеваниям и остеопорозу отягощен не был.

Самому пациенту в отделении проведено расширенное гормональное обследование, патологии гипофиза не выявлено (показатели тиреотропного гормона — 1,17 мМЕ/л, инсулиноподобного фактора роста 1 типа —  182,3 нг/мл, пролактина — 385,9 мЕд/л, в пределах референсных значений). По результатам ранее проведенной МРТ патологических образований поджелудочной железы и надпочечников не обнаружено. Таким образом, из возможных компонентов МЭН1 у мужчины был подтвержден только ПГПТ.

С учетом молодого возраста, тяжелых костных осложнений пациент направлен на хирургическое лечение ПГПТ. В предоперационном периоде назначен цинакальцет с титрацией дозы до 60 мг/сут, однако значимого снижения уровня кальция крови не отмечено (альбумин-скорректированный кальций 3,06 ммоль/л).

В ходе оперативного вмешательства у нижнего полюса левой доли щитовидной железы определялось образование размерами 2,5×1,5×1,0 см. Под контролем возвратно-гортанного нерва выполнено удаление атипично расположенной ОЩЖ, также выполнена ревизия мест типичного расположения ОЩЖ слева, визуализированы левая верхняя и левая нижняя нормальных размеров и структуры. Учитывая неуточненное клиническое значение мутации MEN1, от дальнейшей ревизии было решено воздержаться. На разрезе образование удаленной ОЩЖ аденоматозного вида.

При гистологическом исследовании послеоперационного материала подтверждена аденома ОЩЖ солидного строения из оксифильных клеток, по периферии опухоли обнаружена ткань ОЩЖ с липоматозом.

Уровень интраоперационного ПТГ до удаления составил 211,1 пг/мл, через 15 мин после удаления образования ОЩЖ — 28,9 пг/мл, что свидетельствовало о радикально выполненной операции. Послеоперационный период протекал с клиническими признаками гипокальциемии вследствие синдрома «голодных костей», в связи с чем инициирована терапия препаратами активного метаболита и нативного витамина D (альфакальцидол 1 мкг/сут и колекальциферол 2500 МЕ/сут) и карбоната кальция 1500 мг/сут. Результаты лабораторного обследования в динамике представлены в табл. 3.

**Table:** Таблица 1. Результаты лабораторного обследования при поступлении в стационар

Показатели	Ед. измерения	РИ	Значение
Паратгормон	пг/мл	15–65	208,5
Кальций, скор. на альбумин	ммоль/л	2,15–2,55	2,96
Фосфор крови	ммоль/л	0,74–1,52	0,8
25(ОН)витамин D	пг/мл		46,01
Кальций в суточной моче	ммоль/сут	2,5–7,5	8,22
Остеокальцин	нг/мл	24–70	97,53
С-концевой телопептид коллагена 1 типа	нг/мл	0,1–0,85	1,68
Щелочная фосфатаза	ед/л	40–150	120
Креатинин	мкмоль/л	63–110	78,9
рСКФ EPI	мл/мин/1,73 м2	>90	120

**Table:** Таблица 2. Результаты лабораторного обследования родственников пациента

	Кальций общий, ммоль/л (РИ)	Альбумин, г/л	Кальций, скор. на альбумин	Паратгормон, пмоль/л (РИ 1,45–10,41)	Креатинин, мкмоль/л
Отец, 44 года	2,34 (2,10–2,55)	43	2,28	8,1	97
Мать, 43 года	2,35 (2,10–2,55)	45	2,25	6,7	73
Сестра, 8 лет	2,41 (2,20–2,70)	43	2,35	2,9	48

**Table:** Таблица 3. Результаты лабораторного обследования пациента в послеоперационном периоде

Показатели	Ед. измерения	РИ	Через 1 нед после ПТЭ	Через 4 мес после ПТЭ	Через 6 мес после ПТЭ
Паратгормон	пг/мл	15–65	60,5	33,3	22,51
Кальций, скор. на альбумин	ммоль/л	2,15–2,55	2,07	2,3	2,18
Фосфор крови	ммоль/л	0,74–1,52	-	-	1,16
Кальций в суточной моче	ммоль/сут	2,5–7,5	-	-	6,15
Остеокальцин	нг/мл	24–70	-	-	31,55
С-концевой телопептид коллагена 1 типа	нг/мл	0,1–0,85	-	-	0,61
Щелочная фосфатаза	ед/л	40–150	-	-	83

При повторной госпитализации, спустя 6 мес после хирургического лечения ПГПТ, подтверждена ремиссия заболевания (см. табл. 3). При поступлении пациент отметил хруст и выраженную боль в области левого лучезапястного сустава при подъеме тяжести. По результатам рентгенографии выявлена кистовидно-дистрофическая перестройка дистального эпиметафиза левой локтевой кости с деструкциями от 3,9 мм до 10,7 мм, клинически диагностирован перелом (рис. 6). Отмечена нормализация маркеров костного моделирования, по результатам рентгеновской остеоденситометрии — положительная динамика с приростом МПК в позвоночнике +5,7% (-0,9 SD) и в бедренной кости +7,7% (-2,4 SD), в лучевой кости — без значимых изменений +0,7% (-1,7 SD). Кроме того, при КТ нижних конечностей выявлена положительная динамика в виде преобладания участков остеосклероза в местах кистозной трансформации, признаки консолидации перелома и формирования костной мозоли левой бедренной кости (рис. 7, а, б). Учитывая низко-нормальный уровень кальция крови, а также с целью увеличения минерализации костной ткани увеличена доза альфакальцидола до 1,5 мкг/сут, продолжена терапия колекальциферолом и карбонатом кальция.

Принимая во внимание выраженность костных осложнений заболевания, для определения дальнейшей тактики ведения пациент направлен на консультацию в специализированный травматологический центр. Дополнительно выполнена МРТ коленных суставов, на которой также отмечена частичная репарация участков фиброзно-кистозного остеита (рис. 8).

**Figure:**
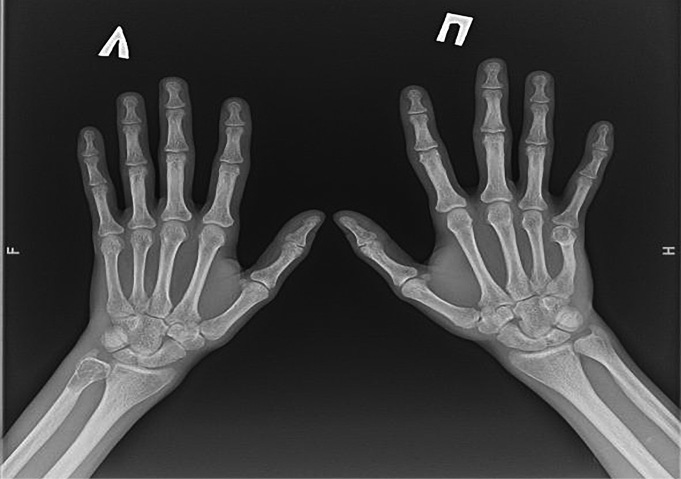
Рисунок 6. Рентгенография кистей с лучезапястными суставами. Диффузное снижение плотности костей, кистовидно-дистрофическая перестройка структуры дистального эпиметафиза левой локтевой кости с деструкциями от 3,9 мм до 10, 7 мм на фоне локальной псевдобуллизации. Справа — ульнарная девиация диафиза 5-й пястной кости с консолидированным переломом в средней трети, деформация с остеодистрофической перестройкой структуры головки и субкапитальной зоны. Перестроечные переломы в стадии консолидации диафизов 3-й и 4-й пястных костей правой кисти по медиальным поверхностям. Формирующиеся кисты в дистальных метаэпифизарных зонах лучевых костей с обеих сторон от 1 до 3 мм, а также в шиловидной зоне дистального эпифиза правой локтевой кости и в телах ладьевидной и полулунной костей правой кисти (0,5–2 мм).

**Figure:**
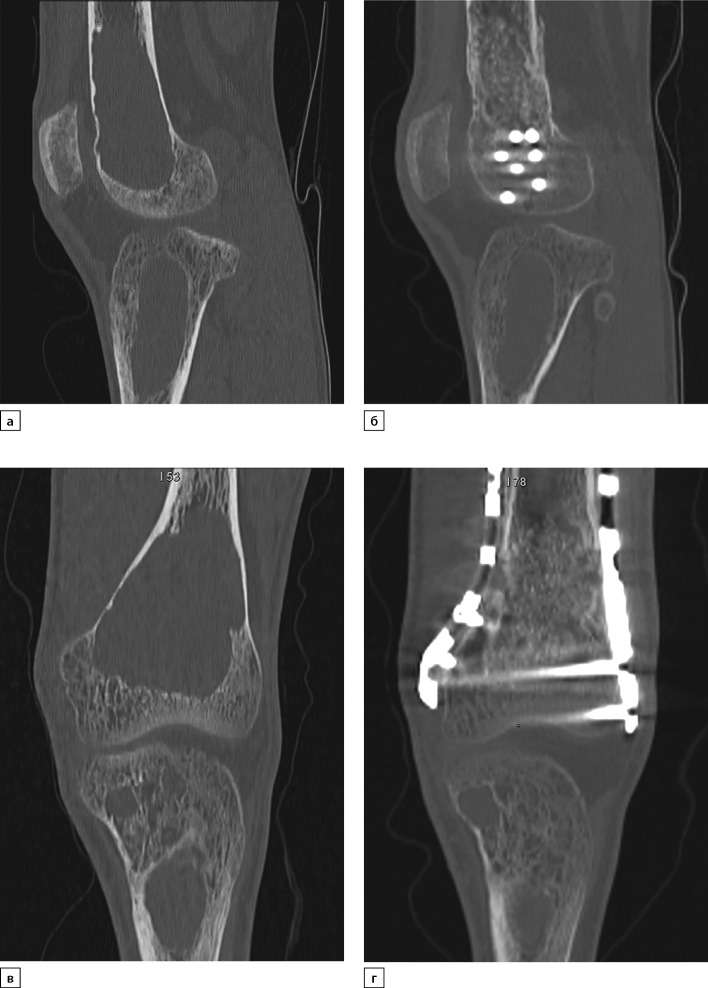
Рисунок 7. Мультиспиральная компьютерная томография левой бедренной кости в динамике: исходно (2020 г., а, в) и через 6 месяцев после паратиреоидэктомии (2021 г., б, г) в сагиттальной и корональной проекциях.

**Figure:**
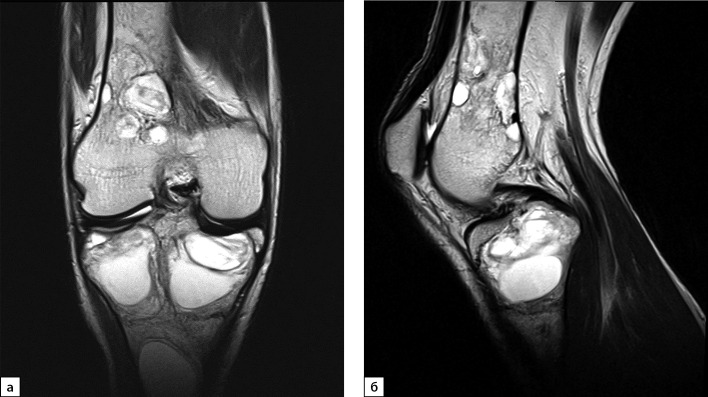
Рисунок 8. Магнитно-резонансная томография правого коленного сустава в режиме Т2, 2021 г. (в корональной (а) и сагиттальной проекциях (б)): МР-картина оссификации и уплотнения содержимого ранее выявленных кист.

## ОБСУЖДЕНИЕ

Особенностью нашего клинического случая является наличие у молодого пациента И. ПГПТ при редкой гетерозиготной мутации в 10 экзоне гена MEN1, осложненного тяжелыми костными нарушениями с множественными патологическими переломами на фоне генерализованного фиброзно-кистозного остеита.

Частота и характер поражения костной ткани при ПГПТ у пациентов с мутациями в гене MEN1 представляет особенный интерес для клиницистов, так как по результатам клинических исследований неоднократно были продемонстрированы более тяжелое течение остеопороза при ПГПТ у пациентов с МЭН1-синдромом по сравнению со спорадическими формами ПГПТ и значительно более медленное восстановление после хирургического лечения ПГПТ [6][9][11–13]. По результатам исследования российской популяции спорадический гиперпаратиреоз сопровождается костными осложнениями в 87% случаев [14]. При ПГПТ в рамках МЭН1 (ПГПТ/МЭН1) снижение МПК определяется у 58–72% таких пациентов [12][15]. В работе A. Silva et al. прирост МПК в течение первого года после радикального лечения ПГПТ у пациентов с МЭН1 наблюдался только в поясничном отделе позвоночника (р=0,2), в то время как у пациентов со спорадической формой заболевания прирост был достигнут как в поясничном отделе позвоночника, так и в бедренной кости, в том числе в ее проксимальном отделе (р<0001; р=0,0004 и р=0,0001 соответственно) [9].

Обсуждается потенциальный вклад мутаций в гене MEN1 в развитие костной патологии. Недавно Crabtree et al. сообщили, что гомозиготная инактивация менина у мышей приводит к дефектам мозгового и лицевого черепа, тогда как гетерозиготная мутация была фенотипически схожа с синдромом МЭН1 у людей [16]. Поскольку кости черепа образуются за счет прямого (внутримембранного) остеогенеза, а не опосредованно через хрящевую ткань (эндохондральную оссификацию), данные результаты предположили возможность участия менина в дифференцировке мультимезенхимальных стволовых клеток в остеобластный ряд [17].

На мышиных моделях продемонстрировано, что менин действительно индуцирует дифференцировку МСК по пути развития остеобластов и ингибирует более позднее созревание уже запрограммированных остеобластов. Данное исследование доказывает модулирующую роль менина как регулятора транскрипции и одновременно супрессора опухоли [17–19].

Существуют важнейшие медиаторы передачи сигналов для костного ремоделирования. К ним относятся BMP-2 (Bone morphogenetic protein-2), Smad1 и Smad5 (ген-специфические факторы транскрипции Similar to Mothers Against Decapentaplegic). Геномные сайты связывания SMAD могут находиться как на проксимальных элементах промотора, так и на энхансерных элементах. Также ключевыми факторами транскрипции остеобластов являются Runx2 (Runt-related transcription factor 2) и JunD (компонент активаторного белка-1 (AP-1), комплекса факторов транскрипции, который играет важную роль в моделировании и ремоделировании скелета) [20][21].

В последующих экспериментах было также показано, что при инактивации менина зафиксирована сверхэкспрессия JunD, повышающая продукцию Runx2, коллагена 1 типа, остеокальцина и щелочной фосфатазы, что в целом отражает высокую метаболическую активность в костной ткани. Из этого были сделаны выводы, что менин подавляет созревание остеобластов частично за счет ингибирования анаболического действия JunD в костной ткани и уравновешивает работу остеобластов и остеокластов [17–19].

В другом исследовании было продемонстрировано, что менин дикого типа напрямую увеличивает транскрипционную активность двух стероидных рецепторов, играющих важную роль в моделировании и ремоделировании костей, — рецептора витамина D (VDR) и рецептора эстрогена α (ERα). В клетках ОЩЖ витамин D подавляет транскрипцию ПТГ и пролиферацию клеток. Снижение активности VDR после утраты менина может способствовать пролиферации клеток ОЩЖ и развитию аденомы [22].

Как у женщин, так и у мужчин костная масса достигает пика в возрасте около 20 лет. В связи с наследственной природой синдрома МЭН1 и молодым возрастом начала патологических процессов нарушение костного метаболизма у этой категории пациентов происходит на самых ранних этапах формирования скелета и в период накопления костной массы [23]. В совокупности всех вышеперечисленных факторов нарушается внутренний контроль за костеобразованием и костной резорбцией. Возникает высокая скорость созревания остеобластов с последующей активацией остеокластов как под влиянием гиперсекреции ПТГ с экспрессией RANKL (Receptor activator of nuclear factor kappa-B ligand), так и, вероятно, в рамках отсутствия инактивирующего действия менина. RANKL синтезируется остеобластами и активированными Т-лимфоцитами и реализует свои эффекты через специфический рецептор RANK на остеокластах и дендритных клетках. Этот эффект нейтрализуется остеопротегерином, который секретируется различными тканями и действует как эндогенный растворимый рецепторный антагонист. Основным источником RANKL являются остеоциты костного матрикса. RANKL и его специфический рецептор RANK — не только ключевые регуляторы ремоделирования костной ткани, они играют также важную роль в иммунобиологических процессах. Остеокластическая резорбция начинает преобладать над минерализацией компактного вещества кости и может приводить к образованию фиброзных кист. Фиброзно-кистозный остеит — позднее костное осложнение при спорадической форме ПГПТ, финальной стадией которого является образование так называемых «бурых опухолей». «Бурые опухоли» не являются новообразованиями, а представляют собой одиночные или множественные остеолитические участки поражения костей в результате быстрой потери костной массы с замещением костного мозга грануляциями и фиброзной тканью [24]. При этом в структуре кости могут быть выявлены зоны некроза с образованием кист с определяющимся уровнем жидкости, кровоизлияний и обызвествлений [25]. Гистологическая структура кистозно-фиброзных изменений при ПГПТ своим строением и клеточным составом может напоминать гигантоклеточные опухоли и некоторые виды сарком [24][26], поэтому обязательна дифференциальная диагностика с другими литическими поражениями скелета, в первую очередь со злокачественными опухолями. В представленном клиническом случае первоначально предполагалось вторичное поражение костей вследствие паранеопластического процесса, в связи с чем проводилась сцинтиграфия костей. Безусловно, выявленные при ней изменения требовали обязательного определения основных показателей минерального обмена, что позволило бы своевременно установить правильный диагноз.

Мутации в гене MEN1 могут приводить к развитию гормонально-активных опухолей других локализаций. В свою очередь, избыточная секреция гормонов гипофиза, надпочечников, щитовидной железы также негативно влияет на костную ткань. У нашего пациента до настоящего времени не выявлено других компонентов наследственного синдрома, что не исключает и спорадический характер мутации MEN1. Тем не менее требуется динамический контроль с оценкой гормональной функции гипофиза и состояния поджелудочной железы. Также, несмотря на отсутствие нарушений фосфорно-кальциевого обмена у родственников 1-й линии родства, рекомендовано их молекулярно-генетическое обследование для выявления бессимптомных носителей мутаций [27] . Чаще всего в пределах одной семьи выявляются однотипные по локализации и характеру мутации [28]. Но каждый клинический вариант МЭН1 не дает достаточной информации для четкого определения уже выявленных мутаций. Следовательно, носители пораженного гена в семействе МЭН1 должны проверяться периодически на наличие других опухолей, нетипичных для конкретного семейства, но типичных для МЭН1 [29].

Вопрос о темпах прироста МПК после успешной паратиреоидэктомии в группе МЭН1-ассоциированного ПГПТ остается открытым, так как данные в литературе ограничены небольшими выборками [30]. M.L. Brandi et al. в своем ретроспективном исследовании сравнили костно-минеральный обмен в до- и послеоперационном периодах в группах пациентов с ПГПТ в рамках подтвержденного МЭН1 (n=133) и спорадической формы (n=47). Независимо от формы заболевания до ПТЭ наблюдались повышенные уровни ПТГ, общего, ионизированного кальция и дезоксипиридинолина, что указывает на активные обменные процессы в костной ткани. При этом пациенты из группы с МЭН1 имели в предоперационном периоде более высокие уровни костной щелочной фосфатазы (BALP), указывающие на более высокую активность остеобластов: ПГПТ при МЭН1 24,1±14,1 мкг/л, спорадический ПГПТ 17,2±7,4 мкг/л, p<0,05). С одной стороны, непосредственно молодой возраст пациентов с МЭН1 может объяснить высокие уровни BALP, однако авторы не исключают прямую роль дефицита менина. При инструментальной оценке, сопоставимо с результатами исследований Wang et al. [31], пациенты с ПГПТ при МЭН1 в сравнении со спорадической формой имели более низкие показатели МПК по Z-критерию в поясничном отделе позвоночника и шейке бедренной кости, при этом обе группы не различались по средним показателям МПК по Т-критерию. Сравнение совокупных данных денситометрии при МЭН1 до и после операции показало, что ПТЭ улучшала прирост костной массы во всех оцениваемых участках. Восстановление костной массы у пациентов с МЭН1 было более выраженным в поясничном отделе позвоночника (трабекулярная кость), чем в шейке бедренной кости (кортикальная кость). Сравнение предоперационных и послеоперационных показателей денситометрии в представленных случаях ПГПТ при МЭН1 показало, что степень улучшения сниженной МПК после ПТЭ сильно варьировала и зависела от времени, прошедшего с момента постановки диагноза до операции. В подгруппе пациентов, имеющих тяжелый остеопороз, наблюдался более ограниченный прирост МПК после ПТЭ, особенно среди лиц молодого возраста. Вероятно, это связано с исходным «недостижением» пика костной массы [32].

Аналогичный нашему пример тяжелых костных осложнений ПГПТ в виде «бурых опухолей» у подростка 17 лет с ПГПТ (уровни ПТГ 896,2 пг/мл, кальция общего 12,5 мг/дл (8,6–10,0), костной щелочной фосфатазы более 200 Ед/л) описан M.E. Atabek et al. [33]. Генетического исследования не проводилось, синдром МЭН1 исключался авторами на основании исследования уровня пролактина и гастрина сыворотки крови, данных ревизии шеи во время хирургического вмешательства — наличие солитарного образования ОЩЖ. Несмотря на отсутствие значимых изменений рентгенологической картины скелета через 2 года после паратиреоидэктомии, повторных низкотравматичных переломов не наблюдалось. Признаки репарации участков кистозной перестройки костей у нашего пациента спустя уже 6 мес после хирургического лечения ПГПТ позволяют надеяться на благоприятный прогноз. Тем не менее, учитывая наличие множественных очагов деструкции костной ткани и угрозу патологического перелома костей, особенно в области проксимального метаэпифиза правой большеберцовой кости, пациент нуждается в дальнейшем оперативном лечении в условиях специализированного травматологического центра.

## ЗАКЛЮЧЕНИЕ

Выявление низкоэнергетических переломов на фоне кистозных образований скелета в молодом возрасте свидетельствует прежде всего о необходимости исключения ПГПТ, в том числе в рамках наследственных форм. Своевременно установленный диагноз и адекватно проведенное хирургическое лечение способствуют улучшению прогноза в отношении жизни больного, хотя выраженный остеопороз и длительная гиперкальциемия в анамнезе требуют обязательной реабилитации и контроля за состоянием костной системы и почечной функции. Данный случай представляет безусловный интерес ввиду клинически агрессивного течения ПГПТ при верифицированной гетерозиготной мутации MEN1. Клиническая значимость мутации c.1609G>T, p.Gly537Cys (rs587780843) заявляется впервые, но требует уточнения. Мы считаем, что сочетание манифестации ПГПТ в молодом возрасте и результаты генетического анализа в большей степени соответствуют проявлениям синдрома МЭН1. Пациент относится к группе риска рецидива ПГПТ после оперативного лечения, что подразумевает более активную тактику наблюдения с регулярным контролем показателей фосфорно-кальциевого обмена. Также необходим регулярный пожизненный скрининг с целью раннего выявления и лечения других компонентов наследственного синдрома. Участие менина в регуляции множества молекулярных путей, в том числе среди клеток остеогенного ряда, диктует необходимость его дальнейшего изучения. В перспективе это может открыть новые мишени для молекулярной терапии компонентов синдрома.

## ДОПОЛНИТЕЛЬНАЯ ИНФОРМАЦИЯ

Источник финансирования. Публикация настоящей работы поддержана грантом РНФ № 20-75-00077 «Характеристика клеток остеогенного ряда в изогенной модельной системе при мутации в гене MEN1».

Конфликт интересов. Авторы декларируют отсутствие явных и  потенциальных конфликтов интересов, связанных с публикацией настоящей статьи.

Участие авторов. Все авторы внесли значимый вклад в подготовку статьи, прочли и одобрили финальную версию перед публикацией.

Согласие пациента. Пациентом была подписана форма информированного согласия на публикацию персональной медицинской информации в обезличенной форме в журнале «Проблемы эндокринологии».
